# The contribution of maternal oral, vaginal, and gut microbiota to the developing offspring gut

**DOI:** 10.1038/s41598-023-40703-7

**Published:** 2023-08-22

**Authors:** Amber L. Russell, Zachary L. McAdams, Erin Donovan, Nicole Seilhamer, Melissa Siegrist, Craig L. Franklin, Aaron C. Ericsson

**Affiliations:** 1 Department of Veterinary Pathobiology, University of Missouri Metagenomics Center, University of Missouri, Columbia, MO 65201 USA; 2 University of Missouri Mutant Mouse Resource and Research Center (MU MMRRC), University of Missouri, Columbia, MO 65201 USA; 3 College of Veterinary Medicine, University of Missouri, Columbia, MO 65211 USA

**Keywords:** Developmental biology, Microbiology

## Abstract

There is limited understanding of how the microbiota colonizing various maternal tissues contribute to the development of the neonatal gut microbiota (GM). To determine the contribution of various maternal microbiotic sites to the offspring microbiota in the upper and lower gastrointestinal tract (GIT) during early life, litters of mice were sacrificed at 7, 9, 10, 11, 12, 14, and 21 days of age, and fecal and ileal samples were collected. Dams were euthanized alongside their pups, and oral, vaginal, ileal, and fecal samples were collected. This was done in parallel using mice with either a low-richness or high-richness microbiota to assess the consistency of findings across multiple microbial compositions. Samples were analyzed using 16S rRNA amplicon sequencing. The compositional similarity between pup and dam samples were used to determine the contribution of each maternal source to the composition of the neonate fecal and ileal samples at each timepoint. As expected, similarity between neonate and maternal feces increased significantly over time. During earlier time-points however, the offspring fecal and ileal microbiotas were closer in composition to the maternal oral microbiota than other maternal sites. Prominent taxa contributed by the maternal oral microbiota to the neonate GM were supplier-dependent and included *Lactobacillus* spp., *Streptococcus* spp., and a member of the *Pasteurellaceae* family. These findings align with the microbial taxa reported in infant microbiotas, highlighting the translatability of mouse models in this regard, as well as the dynamic nature of the GM during early life.

## Introduction

The maturation process of the gut microbiota (GM) is an essential process for life-long health that is defined by the acquisition and colonization of microorganisms in the gut and the subsequent immune system induction that occurs during early life. While emerging evidence suggests that initial colonization of the gut may happen as early as in utero^1,2^, this is controversial^3,4^, and the conventional understanding is that the first bacterial seeding of the gut begins at birth^5^. Regardless of the timing, the initial bacterial colonization of the neonatal gut undoubtedly derives from a maternal source, emphasizing the importance of understanding how vertical transfer is initiated and influenced during continued exposure to maternal microbial sources over the course of GM maturation^6,7^. During the process of maturation, the neonatal GM is especially susceptible to environmental factors capable of inducing persistent effects on the developing GM^8^. Previously identified factors within both human and mouse model populations associated with significant effects on the composition of the neonatal microbiota include mode of delivery^9,10^, breastfeeding^11,12^, and antibiotic exposure^13,14^. These factors have the potential to confound research in human neonates, making characterization of the development and contribution of maternal sources to the neonatal GM difficult^8^.

There is strong agreement across host species regarding the basic characteristics of normal GM maturation that correspond with physiological changes occurring in the gastrointestinal tract^15,16^. During early life, the neonatal GM of vaginally born, breastfed infants and nursing mouse pups alike is dominated by members of the *Enterobacteriaceae* and *Lactobacillaceae* families^7,17^, which are transferred from mother during parturition and subsequently colonize the neonatal GM due to the high oxygen availability in the gastrointestinal tract relative to adults^15,18^. Next, as lumenal oxygen tension decreases, and breastfeeding continues, *Bifidobacteriaceae* and *Clostridiaceae* proliferate until weaning, presumably due to their role in metabolism of milk oligosaccharides and facultative anaerobic capabilities^19,20^. With the introduction of solid foods^16^, a more diverse and adult-like GM develops, with *Bacteroidaceae*, *Lachnospiraceae*, and *Ruminococcaceae* being dominant families of the mature GM^8,16,21^, and the maturation process can be considered complete once the GM has reached a richness and composition consistent with that of the maternal feces^6,8^.

There is limited knowledge regarding the contribution of different maternal source microbiotas in the neonatal mouse GM. Literature describing the vertical transfer and maturation of the human GM has relied on noninvasive sampling, and largely ignored the upper gastrointestinal tract (GIT)^6,7,22^. The few studies of the developing murine GM that included samples of the neonatal ileal microbiome found compositional and functional differences compared to the neonate fecal composition, but the maternal source of ileal microbes was not investigated^23,24^. Considering the abundance of gut-associated lymphoid tissue in the ileum, and production of short-chain fatty acids in the colon, microbial populations at both sites are physiologically relevant.

To address these knowledge gaps, we characterized the neonatal fecal and ileal microbiota of entire litters of mice at multiple pre-weaning time-points, alongside the maternal fecal, ileal, vaginal, and oral microbiotas using targeted amplicon sequencing. Bray–Curtis distances were utilized to assess the relative compositional similarity between neonatal and maternal samples. The mean contribution of maternal sources on the fecal and ileal neonatal microbiota at each timepoint were determined using SourceTracker2.

Considering the significant differences in richness and beta-diversity among specific pathogen-free (SPF) microbiota from different suppliers, the entire experimental design was replicated in two separate colonies of CD-1 mice, each harboring a different supplier-origin gut microbiome (GM)^25^, designated GM1 (originating from the Jackson Labs) and GM4 (originating from Envigo). GM1 is of lower richness than GM4, but they are both naturally occurring, complex, SPF microbiomes.

## Results

### Phylum-level composition changes rapidly during GM development

**Figure 1:**
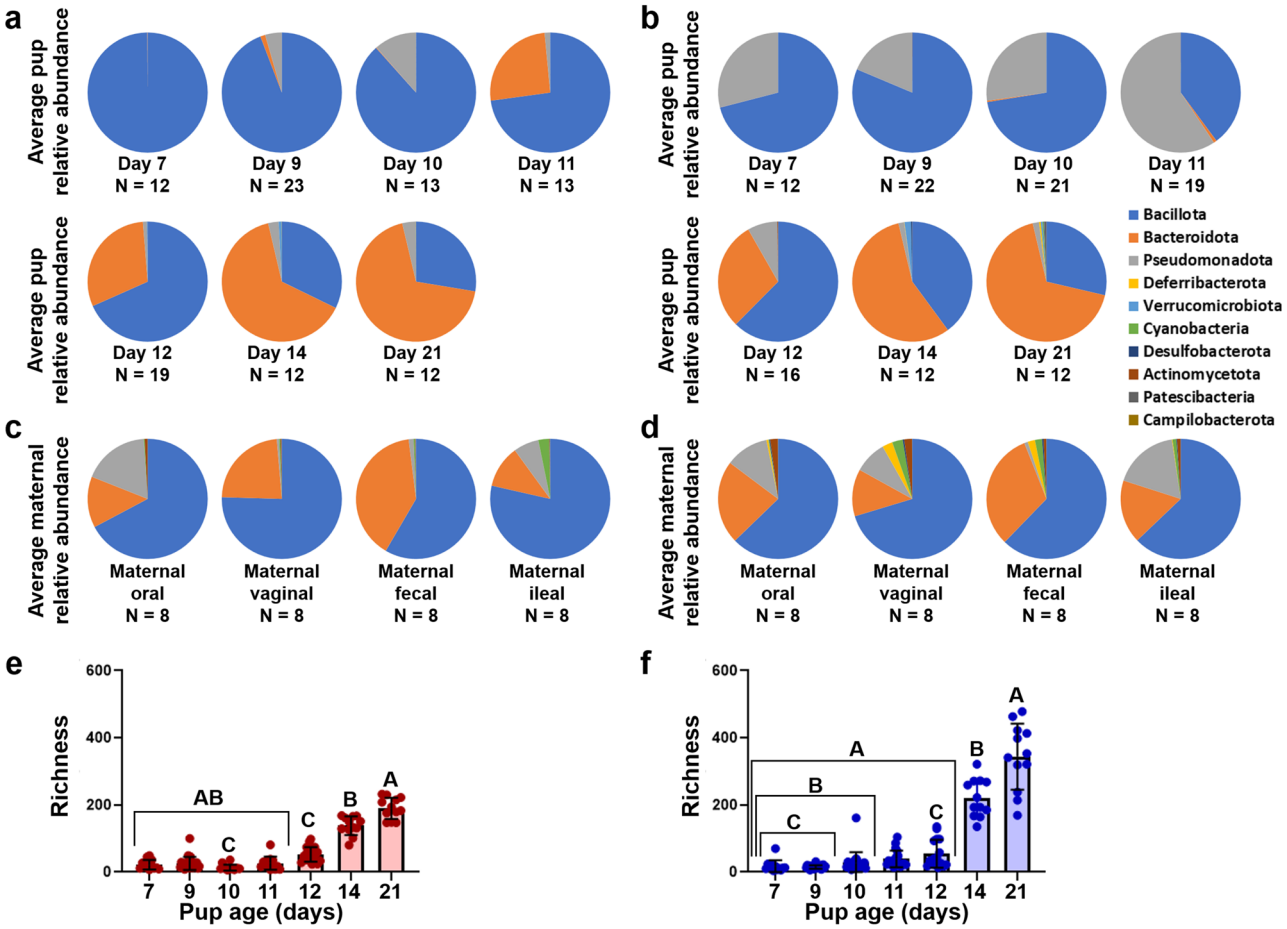
Fecal *Bacteroidota* relative abundance and alpha diversity are greater in older pups. (**a–d**) Circle graphs depicting average relative abundance of samples. Figure key located on right side of panel. (**a,b**) Pup fecal samples grouped by timepoint from (**a**) GM1 and (**b**) GM4 mice. (**c,d**) Maternal tissue samples groups by sample tissue type for (**c**) GM1 and (**d**) GM4 mice. (**e,f**) Bar charts depicting richness of samples with bars representing group averages and error bars representing standard error of mean (SEM). Each symbol represents an individual sample. (**e**) Average GM1 pup fecal alpha diversity grouped by timepoint. (**f**) Average GM4 pup fecal alpha diversity grouped by timepoint. Statistics were calculated using ANOVA on ranks within each GM, like letters within a graph denote a significant difference in pair wise comparisons using Dun’s post hoc.

Neonate feces demonstrated incrementally lower relative abundance (RA) of *Bacillota* and higher RA of *Bacteroidota* at each subsequent timepoint (Fig. 1a,b), with the latter at substantially higher proportions from day 11 or 12 onward. Prior to day 12, neonate feces harboring the high richness gut microbiota (GM) composition, GM4, contained high RA of *Pseudomonadota*, while this phylum was present at a much lower RA in neonate feces of mice harboring the low richness GM composition, GM1. The same difference was observed in the neonate ileum (Supplemental Fig. 1a,b). Maternal vaginal and ileal samples from GM1 (Fig. 1c) contained lower proportions of *Pseudomonadota* than samples from GM4 (Fig. 1d). There was also a greater number of bacterial phyla within vaginal and fecal samples from GM4 dams. The vaginal and fecal microbiota of GM4 maternal samples contained populations of *Bacillota*, *Bacteroidota*, *Deferribacterota*, *Verrucomicrobiota*, *Cyanobacteria*, and *Actinomycetota* at proportions of 1% or greater, while GM1 samples contained only *Bacillota*, *Bacteroidota*, and *Pseudomonadota* at proportions of 1% or greater.

An ANOVA on ranks test was used to identify differences in richness within each GM, defined as the number of unique ASVs within a sample. As expected, the richness of neonate fecal samples was significantly higher at later timepoints, however within ileal samples, age did not have a significant main effect on richness in either GM. For GM1 mice, fecal richness at day 14 and day 21 was significantly higher than samples from day 7 through day 11 (Fig. 1e), while ileal richness had no clear pattern based on timepoint (Supplemental Fig. 1). A similar effect was observed in GM4 neonate richness, where fecal richness at day 21 was significantly higher than day 7 through 12 (Fig. 1f), and no difference in richness was detected in the ileum (Supplemental Fig. 1).

### Evidence of GM-specific vertical transfer

**Figure 2:**
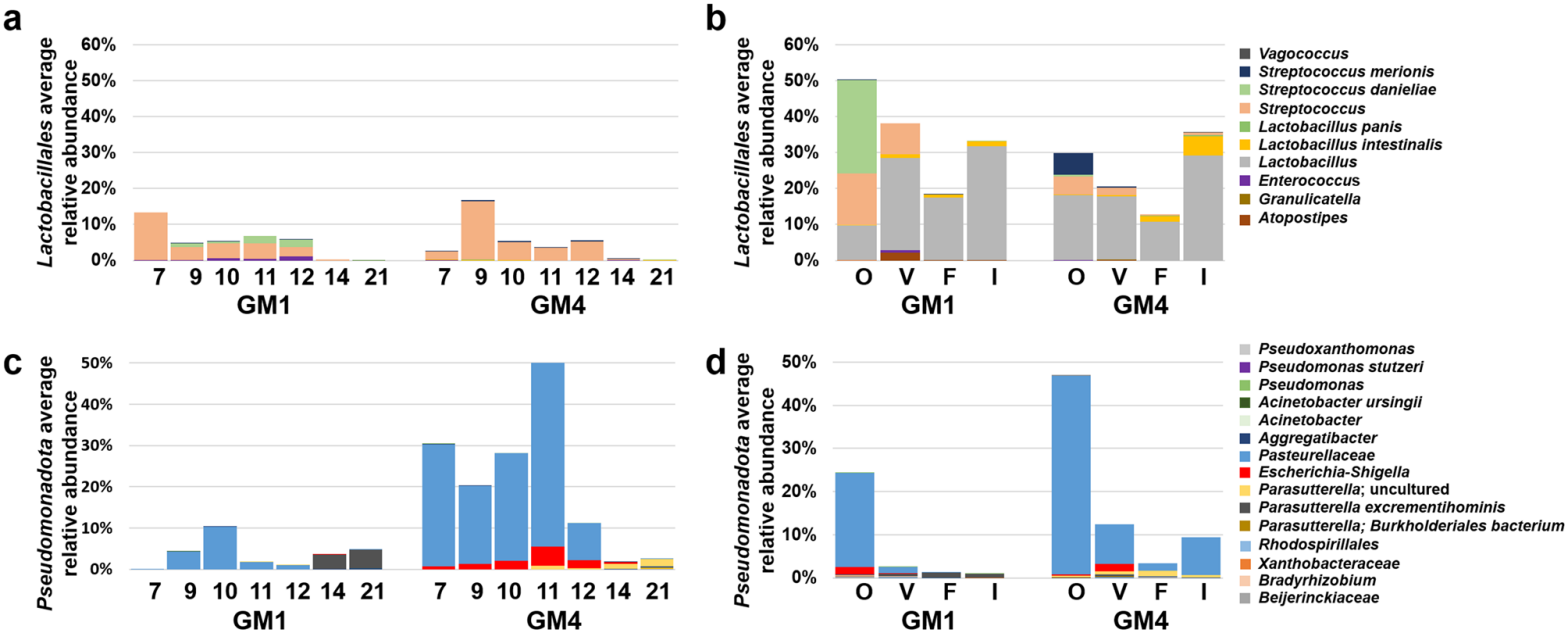
GM4 Pup feces harbors a higher proportion of *Pseudomonadota* species than GM1. (**a,b**) Bar charts depicting the average proportion of taxa annotated to *Lactobacillales* in study samples. Figure key is located on the right side of panel. (**a**) Pup fecal samples grouped by GM and age of pups. (**b**) Maternal samples grouped by GM and tissue type. (**c,d**) Bar charts depicting the average proportion of ASVs annotated to *Pseudomonadota* in study samples. Bar charts depicting the average proportion of taxa annotated to *Pseudomonadota* in study samples. Figure key is located on the right side of panel. (**c**) Pup fecal samples grouped by GM and age of pups. (**d**) Maternal samples grouped by GM and tissue type.

The dominant phyla of the neonatal GM during the earlier stages of development consistent between GM1 and GM4 *were Bacillota* and *Pseudomonadota* (Fig. 1). To better resolve the taxonomic differences between GM1 and GM4 within these phyla, we determined the RA of ASVs annotated to the phylum *Pseudomonadota* and the order *Lactobacillales*, a dominant order of the mouse fecal, oral, and vaginal microbiota within the phylum *Bacillota*^26–28^. The RA of *Lactobacillales* was lower at day 21 compared to day 7 through day 12 in both GM1 and GM4 neonate fecal samples (*p* = 001, Fig. 2a). Regarding the different maternal sites (Fig. 2b), the RA of *Lactobacillales* was variable in both GMs, with statistical difference between sampling sites for GM1 (H = 17.91, *p* = 0.001), but not for GM4 (H = 7.34, *p* = 0.12). The oral microbiota contained GM-specific species of *Streptococcus*, including *S. danieliae* in GM1, and *S. merionis* in GM4, and this difference was reflected in the neonatal feces. For neonate fecal ASVs annotated to the phyla *Pseudomonadota*, an ASV annotated to the family *Pasteurellaceae* was dominant in both GMs, particularly in GM4, reaching a mean RA greater than 50% at day 11 before declining significantly at all later time-points (Fig. 2c). A similar trend was observed for *Escherichia-Shigella* in neonate feces*,* wherein the RA was higher within GM4 at day 12 compared to earlier timepoints (day 7: *p* = 0.04, day 9: *p* = 0.007). On day 14 and 21 in neonate feces, *Pasteurellaceae* and *Escherichia-Shigella* were largely replaced as the dominant ASVs by *Parasutterella excrementihominis* in GM1, and an unspeciated *Parasutterella* in GM4 (Fig. 2c). A high RA of the dominant *Pasteurellaceae* ASV was detected in maternal oral samples, and was significantly more abundant relative to maternal fecal samples (H = 14.313, *p* = 0.016).

The neonatal ileum was distinct from feces but also reflected several trends seen in the neonate feces, including GM1-specific growth of *S. danieliae* (Supplemental Fig. 2). Similarly, *Pasteurellaceae* represented the dominant *Pseudomonadota* within the neonatal ileum, showing a similar peak RA approaching 50% in the GM4 neonate ileum at day 11 (Supplemental Fig. 2). Collectively, these data suggest that dominant taxonomies are shared between the maternal oral cavity and neonate mouse gut.

Stacked bar charts representing the most abundant genera within maternal tissue (Supplemental Fig. 3) were used to visualize the overall composition of each tissue and compositional differences between maternal sources. Results showed similar genera shared between GM1 and GM4, however these genera were at different RA depending on the GM. Similar bar charts representing the most abundant genera were also made to visualize composition development of the neonate fecal and ileal communities (Supplemental Fig. 4). Results corroborate differences in dominant genera between GM1 and GM4 neonate samples as early as day 7.

### Similarity to maternal tissue depends on neonatal GI sample location

**Figure 3:**
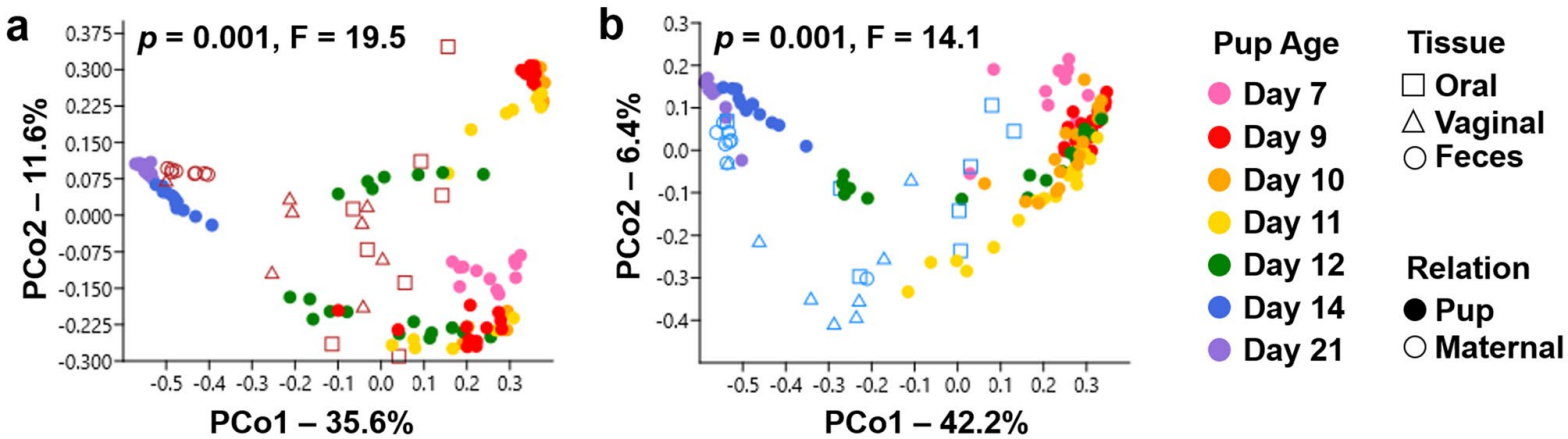
Neonatal fecal similarity to maternal tissues shifts dramatically with time in both GMs. Principal coordinate analysis (PCoA) plots depicting beta diversity using Bray–Curtis distances. Figure key to the right side of panel. (**a,b**) Pup fecal samples with maternal oral, vaginal, and fecal samples from groups (**a**) GM1 (**b**) GM4. Statistics were calculated using a one-factor PERMANOVA for each graph.

Principal coordinate analysis (PCoA) was used to visualize the beta diversity of neonatal and maternal samples within each GM composition. Bray–Curtis PCoA was used to visualize the compositional shift of neonate fecal samples at each timepoint in relation to the composition of maternal oral, vaginal, and fecal samples. In both GM1 (Fig. 3a) and GM4 (Fig. 3b), PCoA resulted in clustering of pup feces from earlier timepoints (day 7 to day 12) with maternal oral (and to a lesser degree) vaginal samples. In contrast, neonate fecal sample collected at days 14 and 21 clustered closely with maternal fecal samples.

An additional Bray–Curtis PCoA was utilized to visualize neonate ileal samples at each timepoint in comparison to maternal oral, vaginal, and ileal microbial compositions. Within neonate ileal samples, there was less clustering according to timepoint, and less separation from maternal samples. Within both GM1 and GM4 (Supplemental Fig. 5), maternal oral and ileal samples overlapped with neonate ileal samples along the first two principal coordinates while maternal vaginal samples were at the periphery of this overlapping cluster. Collectively, these data suggest that the offspring GM does not mature until the third week of life, and that the immature GM is more similar in composition to the maternal oral microbiota than the vaginal microbiota.

### The maternal vaginal contribution to neonatal SPF microbiota is negligible

**Figure 4:**
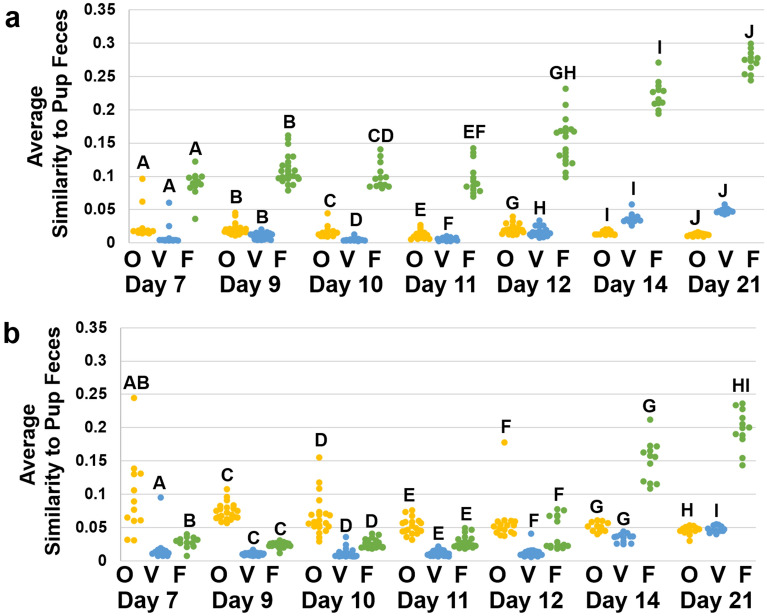
Fecal similarity increases over time, but similarity to maternal oral microbiome is GM dependent. (**a,b**) Dot plots and bar graphs depicting Bray–Curtis similarity of pup feces to maternal tissue. (**a**) Average Bray–Curtis distance of maternal samples to pup feces in GM1. Each dot represents the average Bray–Curtis distance of one pup fecal sample to all maternal samples of that tissue type in GM1. (**b**) Average Bray–Curtis distance of maternal samples to pup feces in GM4. Each dot represents the average Bray–Curtis distance of one pup fecal sample to all maternal samples of that tissue type in GM4. Statistics were calculated within each GM using a Two-way ANOVA on tissue type and age of pups. Matching letters denote significant pairwise differences detected during a Holm-Sidak post hoc analysis. *O* Oral, *V* Vaginal, *F* Fecal.

To control for dam-to-dam variation, we calculated the average Bray–Curtis compositional similarity values (1 − Bray–Curtis dissimilarity value) comparing neonate fecal samples and maternal fecal, oral, and vaginal sample for mice harboring GM1 and GM4. The Bray–Curtis similarity values for neonate fecal samples were calculated by comparing the compositional similarity of an individual neonate fecal sample to all maternal samples (n = 8) for each maternal site (oral, vaginal, fecal). The average Bray–Curtis similarity value for each maternal site was then calculated for each neonate fecal sample and utilized for analysis. A three-way ANOVA was used to identify main effects and interactions between GM, age of pups, and maternal sampling site. There was a significant main effect of all factors, and significant interactions between all three factors analyzed (F = 2.38, *p* = 0.005). In order to analyze pairwise comparison between samples within each GM, data were stratified by GM and a two-way ANOVA was utilized. Within GM1, neonate feces had the highest similarity to maternal fecal samples at all timepoints compared to oral and vaginal samples (Fig. 4a). The similarity between pup feces and the maternal oral and vaginal microbiotas was much lower and favored similarity to the oral microbiota during early life. Within GM4, maternal oral samples had significantly higher similarity to pup feces than maternal vaginal or fecal samples from day 7 to day 12 (Fig. 4b). By day 14, similarity of offspring feces to maternal feces was significantly higher than similarity to oral (*p* < 0.001, t = 12.48) or vaginal samples (*p* < 0.001, t = 14.64), which remained true on day 21.

Bray–Curtis similarities between neonate ileal samples and offspring-dam matched maternal samples were also compared, during the period between days 9 and 12 for each maternal site. A three-way ANOVA found an overall main effect of age of pups (F = 12.30, *p* < 0.001) and maternal tissue (F = 13.48, *p* < 0.001), as well as interactions between all three factors (F = 6.20, *p* < 0.001). Two-way ANOVA stratified by GM found a significant difference between maternal tissue type within GM1 on day 10 and 11 (Supplemental Fig. 6a). Within GM4 ileal samples, there was a significant difference between maternal tissue type at each timepoint (Supplemental Fig. 6b). While similarity to maternal sites in each GM varied from day to day during this period, the highest similarity was most often to maternal oral or ileal samples.

Next, we utilized Spearman correlation analyses to determine the correlation between maternal tissue similarity to both the neonatal upper and lower GIT. Neonates that had both fecal and ileal samples collected were used for this analysis (n = 146). There was a significant positive association between maternal oral and neonatal GIT similarity, while maternal vaginal similarity to neonatal fecal and ileal samples did not correlate significantly (Supplemental Fig. 6c–e).

Neither maternal vaginal nor maternal fecal samples correlated with neonatal GIT samples in terms of similarity. These data suggest that those mice in which the maternal oral microbiota is most represented in the neonatal ileum, are expected to have the highest similarity between maternal oral and neonatal fecal microbiota as well.

### The neonatal hindgut is dominated by the maternal oral microbiome

**Figure 5:**
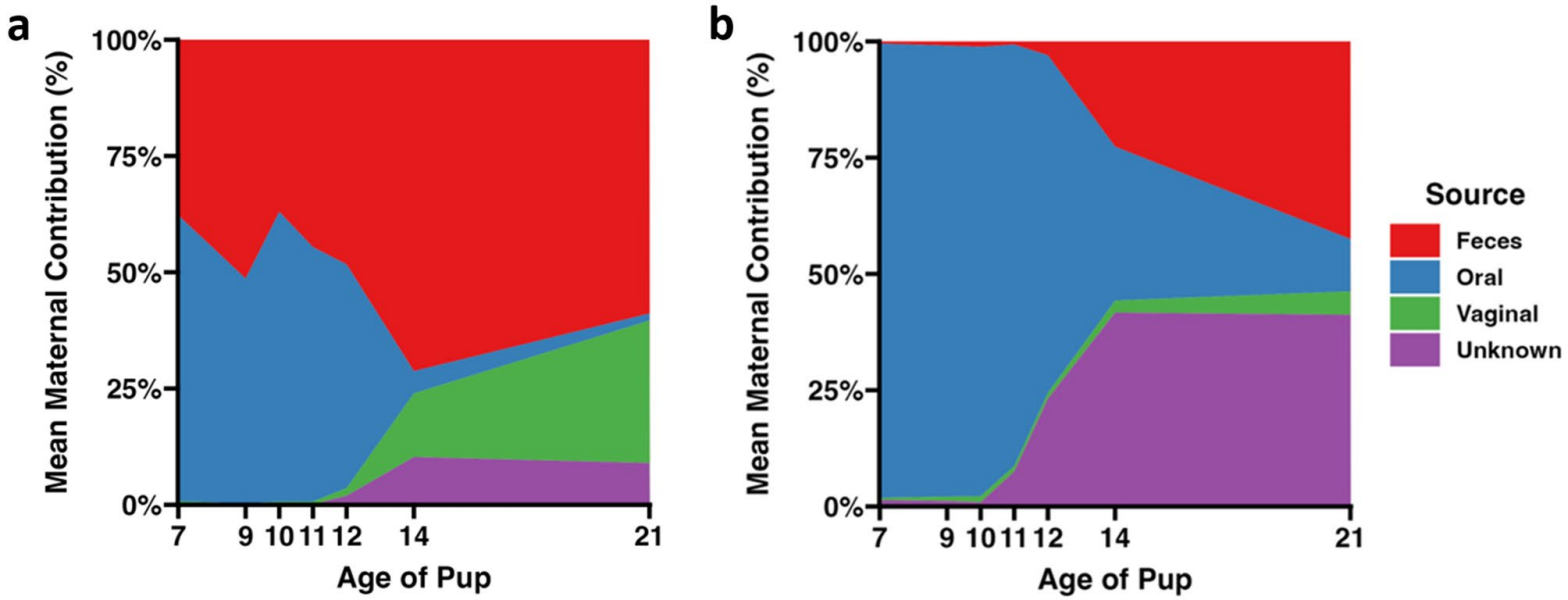
Unique maternal vaginal ASVs were not incorporated into pup fecal microbiota. Area graphs depicting the proportion of amplicon sequence variants (ASV)s found in the pup fecal core microbiota at each timepoint, that are also found in maternal tissue core microbiota. Figure key is located to the right of the panel. (**a**) GM1 pup fecal and (**b**) GM4 pup fecal core microbiota.

Lastly, data were analyzed using SourceTracker software, with each maternal and offspring site identified as source and sink, respectively. SourceTracker uses a Bayesian model to accurately predict the relative contribution of multiple sources to a given sink site^29^. Using this approach, the fecal microbiome of neonatal SPF mice originates primarily from the maternal oral cavity during early life, with lower to negligible colonization by maternal fecal microbes during that time-point (Fig. 5). Shortly before the second week of life, there is increased representation of microbes found in maternal fecal or vaginal samples, or not found at all in any maternal site. Interestingly, when the contribution of the maternal ileal, oral, or vaginal microbiomes to the neonatal ileum are assessed, the data clearly show a dominant contribution of the maternal ileum to the offspring ileum, across time and in both microbiomes (Supplemental Fig. 7). Collectively, these results are in agreement with earlier analyses indicating a substantial contribution of the maternal oral microbiome to the fecal microbiome of SPF mice during the first 2 weeks of life, followed by maturation and approximation of the maternal fecal microbiome during the third week of life.

## Discussion

Our results indicate that the GM of neonatal mice rapidly changes during development to approach the maternal GM in composition and diversity by 3 weeks of age (Fig. 1). Within feces, proportions of *Bacillota* and *Pseudomonadota* decrease, while proportions of *Bacteroidota* increase throughout maturation. These changes in phylum level RA are consistent with previous findings indicative of the diversification of the GM population seen in both humans and mice at weaning^30^. The expansion of *Bacteroidota* is correlated with the onset of consumption of plant derived carbohydrates found in solid foods in both humans and mice^16^, likely explaining the increase seen in this study. The decline in the proportion of *Pseudomonadota* and *Bacillota* as pups age may be due to the shift from an aerobic to an anaerobic environment within the intestines due to the decline in oxygen availability as pups age, causing a compositional shift favoring bacteria that are able to utilize anerobic respiration^31^. The maturation of the GM is further exemplified in these pups by the steady increase in fecal alpha diversity over time, as has been reported previously during early development in both species^7,8,32,33^.

The RA of the phylum *Pseudomonadota* was strikingly different between GM1 and GM4 in both neonatal fecal and ileal samples (Fig. 2, Supplemental Fig. 2). Specifically, GM4 mice harbored higher proportions of *Pseudomonadota* compared to GM1 mice, as two ASVs annotated to *Pasteurellaceae* and one *Escherichia-Shigella* ASV were enriched in GM4 compared to GM1 neonatal feces. Interestingly, maternal *Escherichia-Shigella* was found in highest proportions in oral samples in GM1 but in vaginal samples in GM4. We also found that the RA of *Pseudomonadota* changes in the neonatal GM over time, but *Lactobacillales* colonization appears less dynamic during the maturation of the GM. This may be due to intermittent presence of *Pseudomonadota* in the maternal milk microbiota^12,34^ and the ability of *Pseudomonadota* to metabolize milk oligosaccharides^35^, while the decline in *Pseudomonadota* in neonatal mice has been associated with increased IgA production as neonates age^36^. In contrast, *Lactobacillales* are lactic acid-producing bacteria that utilize carbohydrates and colonize the gut throughout infancy and adulthood and can survive in both anerobic and aerobic conditions^37^. The higher proportions of *Streptococcus danieliae* in GM1 and *Streptococcus merionis* in GM4 in the neonatal GM were vertically transferred from the maternal oral microbiota. These results suggest that different background GMs are predisposed to harboring not only different proportions of bacteria, but also experience species-specific GM vertical transfer of these populations. Bar charts characterizing the dominant genera of maternal oral, vaginal, fecal, and ileal samples (Supplemental Fig. 3) and neonate fecal and ileal samples (Supplemental Fig. 4) provide further support of these findings.

As pups age, both fecal and ileal GM beta diversity became increasingly similar to maternal fecal and ileal samples respectively, as well as inter-individual similarity within pups (Fig. 3, Supplemental Fig. 3). This increase in similarity is expected, and has repeatedly been reported in the literature of both human and mouse model as a hallmark of GM^17,38^. The contribution of maternal microbial sites to the neonatal GM varies between different SPF GMs. In terms of beta diversity, the similarity of offspring feces to oral maternal samples remained stable overtime, however in GM4, this similarity was significantly higher than fecal maternal similarity until day 14. This provides evidence that maternal tissue contribution varies between GM compositions, and we may thus infer that vertical transfer from maternal microbial sites is mediated in part by the composition of the various source microbiotas. The similarity of offspring ileum to maternal tissue samples was inconsistent over time, but findings suggest the same trends seen in the feces.

Analysis of beta diversity similarity (Fig. 4, Supplemental Fig. 4) and SourceTracker analysis used to determine the relative contribution of maternal oral, vaginal, fecal, and ileal microbiota to the neonatal fecal and ileal microbiota (Fig. 5, Supplemental Fig. 5) revealed high similarity between pup feces and maternal oral samples in early life, with increasing similarity to maternal gastrointestinal samples as pups age. These results are consistent with recent studies, in which the neonatal GM was colonized least by maternal vaginal bacteria relative to other maternal sources^6^. As such, this study provides further evidence of conserved events between humans and mice during the maternal transfer of microbes and maturation of the offspring GM.

Offspring fecal samples from mice with each GM were originally collected at day 7, 14, and 21 to examine changes in the fecal microbiota over time. We found a large compositional shift between day 7 and day 14 in which the neonatal mouse fecal microbiota underwent a dramatic change in composition. To examine this compositional shift in greater detail, we focused on days 9, 10, 11 and 12 to examine the bacterial composition of pup ileum in relation to different maternal sites during this period of transition. Although we do not have ileal data for all timepoints, we found the data pertinent to include in this analysis for a more complete understanding of neonatal GM development.

The results of this study are overall consistent with recent literature regarding GM development and vertical transfer of various maternal microbiota sources. Previous maternal transfer studies have focused on human rather than mice, as such our study provides novel information regarding the effect of multiple microbiota sources on GM development in mice. By utilizing the differing compositions of GM1 and GM4, this study was able to model some of the GM variation seen between individuals in human studies, thus allowing us to find significant differences in the contribution of various maternal bacteria on the pup GM composition throughout development. These results support the use of the mouse as an appropriate model for GM development and highlight the importance of utilizing a study design with more than one GM composition.

Limitations of the study include the inclusion of only two gut regions in offspring, and the lack of maternal milk as an additional source of microbes. The selection of these sample sites (and sample collection time-points) was based on a combination of financial and logistic considerations. Similarly, while we attempted to assess the transfer of maternal microbes to murine offspring in multiple supplier-origin SPF microbiomes, there are additional suppliers providing mice with their own distinct microbiomes that were not included in the current study. Other SPF mouse microbiomes present in mice used in U.S. biomedical research (i.e., Charles River Lab and Taconic) are intermediate between GM1 (Jackson-origin) and GM4 (Envigo-origin) in terms of both alpha- and beta-diversity^39^, and we speculate that the overall kinetics and contribution of maternal sources to offspring would follow a similar pattern in those communities. Regarding the technical components, no positive or negative sequencing controls were processed and sequenced alongside the experimental samples. Ideally, such controls are used to validate the ability of extraction protocols and PCR primers to retrieve and amplify bacterial DNA with minimal bias, and to identify and remove the sequences from contaminating DNA present in reagents. We have previously validated our DNA extraction and sequencing pipeline using mock microbial communities^40^, and used reliable kit-based procedures for all processes. Given the high read counts obtained for all samples, including relative low biomass samples such as the vaginal and oral swabs, we are confident in our findings and overall conclusions.

In conclusion, SPF mouse microbiotas undergo a dynamic and somewhat characteristic maturation process, culminating by roughly two to three weeks of age. Prior to that, the neonatal GM is more similar in composition to the maternal oral microbiota, as opposed to the vaginal and fecal microbiotas. Additionally, the maternal source microbiota that is transferred during GM development is dependent on the specific SPF microbiota. Further studies are needed to expand our knowledge regarding the effect of these developmental exposures on host development, and if additional maternal bacterial sources, such as the skin, contribute significantly to GM development.

## Methods

### Animals

All experiments described in this manuscript were performed in accordance with the guidelines outlined in the Guide for the Care and Use of Laboratory Animals, and were approved by the Institutional Animal Care and Use Committee (IACUC) of the University of Missouri (protocol 9587). This study was also reported in accordance with ARRIVE guidelines. Outbred CD-1 mice maintained at the University of Missouri harboring either a low richness background GM originating from The Jackson Laboratory, designated GM1, or a high richness background GM originating from Envigo, designated GM4. A detailed description of these colonies has been previously published^25^. 16 female mice (GM1: n = 8, GM4: n = 8) were bred and their subsequent pups were allowed to age until either day of age 9 (GM1: n = 23, GM4: n = 22), 10 (GM1: n = 13, GM4: n = 21), 11 (GM1: n = 13, GM4: n = 19), or 12 (GM1: n = 19, GM4: n = 16). Two dams and their subsequent pups were euthanized at each timepoint chosen at random, and samples were collected immediately after euthanasia. An additional 12 pup fecal samples were also collected at 7, 14, and 21, days of age from colony pups from both GM backgrounds for comparison to the dam-pup matched samples. Mice were housed at Discovery Ridge in Columbia, MO under barrier condition in microisolator cages on Thoren ventilated racks under a 14:10 light/dark cycle. Each cage contained pelleted paper bedding with nestlets and received ad libitum access to irradiated Breeder diet 5053 rodent chow and acidified, autoclaved water.

### Sample collection

All maternal samples were collected *post mortem* at days 9, 10, 11, and 12. Oral and vaginal swabs were used to collected oral and vaginal microbiota samples respectively, by inserting a cotton swab into the designated orifice and rotating the swab. Swab samples were then collected into 2 mL round-bottom tubes. Maternal ileal samples were collected by excising roughly 4 cm of ileum proximal to the ileocecal junction and rinsing the luminal contents of the sample into a 2 mL round-bottom tube using sterile PBS. For maternal fecal samples, the two most distal fecal pellets in the rectum or distal colon were collected and placed in 2 mL round-bottom tubes with a 0.5 cm-diameter stainless steel ball bearing for homogenization of sample. Pup feces were collected as described for dams at days 7, 9, 10, 11, 12, 14, and 21 of age. Pup ileal samples were collected at days 9, 10, 11, and 12 of age by excising 2 cm of the ileum proximal to the ileocecal junction and collecting into a 2 mL round-bottom tube with a 0.5 cm-diameter stainless steel ball bearing due to the small size of the ileal lumen. All samples were placed on ice immediately following collection and samples were stored in a  − 80 °C freezer until DNA was extracted.

### DNA extraction

All sample tissue DNA was extracted using PowerFecal Pro kits (Qiagen) according to manufacturer’s protocol, with the exception that samples were homogenized using a TissueLyser II (Qiagen) for 10 min at 30/sec, in lieu of a vortex adapter as described by PowerFecal Pro kit instructions. DNA yields were quantified by fluorometry via the quant-iT BR dsDNA reagent kits (Invitrogen) and normalized to a consistent concentration and volume prior to submission for downstream processing.

### 16S rRNA library preparation and sequencing

Tissue sample DNA was processed at the University of Missouri Genomics Technology Core. Bacterial 16S rRNA amplicons were constructed via amplification of the V4 region of the 16S rRNA gene with the universal primer set (U515F/806R) and flanked by Illumina standard adapter sequences as in a method previously described elsewhere^41,42^. Dual-indexed forward and reverse primers were used in all sample reactions. PCR was initiated in 50 µL reactions containing 100 ng metagenomic DNA, primers (0.2 µM each), dNTPs (200 µM each), and Phusion high-fidelity DNA polymerase (1U, Thermo Fisher). Amplification parameters used were as followed: 98 °C (3 min) + [98 °C (15 s) + 50 °C (30 s) + 72 °C (30 s)] × 25 cycles + 72 °C (7 min). Amplicon pools of 5 µL/reaction were combined, thoroughly homogenized, and then purified with addition of Axygen Axyprep MagPCR clean-up to an amplicon volume of 50 µL. Amplicons were then incubated for 15 min at room temperature and received multiple washes of 80% ethanol. Post-wash, the dried pellet was resuspended in 32.5 µL EB buffer (Qiagen), incubated at room temperature for 2 min, and then placed on a magnetic stand for five minutes. The final amplicon pool was then evaluated using quant-IT HS dsDNA reagent kits and diluted according to the Illumina standard protocol for sequencing of 2 × 250 bp paired-end reads. Amplicon pools were then sequenced on the MiSeq instrument.

### Informatics analysis

DNA sequences were assembled and annotated at the MU Bioinformatics and Analytics Core. The primer set was designed to match the 5ʹ end of both the forward and reverse amplicon reads. Cutadapt4 (version 2021.8.0; https://github.com/marcelm/cutadapt) was then used to remove the primer from the 5ʹ end of the forward read, and then if found, remove the reverse complement of the primer to the reverse read from the forward read. Therefore, a forward read could be trimmed at both ends if the insert were shorter than the length of the amplicon. The same method was then utilized for reverse reads, but with the primers in the opposite roles. Read pairs were then rejected if one read or the other did not match a 5ʹ primer, and the allowed error-rate was 0.1. Two passes over each read count were made to ensure removal of the second primer, and a minimal overlap of three bp with the 3ʹ end of the primer was required for removal.

The QIIME2 DADA2 plugin (version 1.18.0) was utilized to denoise, de-replicate, and count amplicon sequence variants (ASVs). The following parameters were incorporated: (1) forward and reverse reads were truncated to 150 bases, (2) forward and reverse reads with an expected error higher than 2.0 were ignored, and (3) Chimeras were detected using the “consensus” method and then removed. Python version 3.8.10 and Biom version 2.1.10 were used in QIIME2. Taxonomies were assigned to each of the final sequences using the Silva.v138 database, utilizing the classify-sklearn procedure. An average sequencing depth of 84,706 reads was achieved. No normalization or rarefication of reads were utilized.

### Statistics and graphing

To calculate significant changes in alpha diversity and RA of pup fecal and pup ileal samples, we utilized ANOVA on ranks within each GM, due to a lack of normality. Community richness, the number of unique ASVs within a sample, using PAST software^43^.

An ANOVA on ranks was performed using SigmaPlot 14.0 with a Dunn’s post hoc analysis for pairwise comparisons. Significant differences in RA of *Lactobacillales* and *Pseudomonadota* ASVs between neonatal samples at different timepoints, as well as between maternal microbial tissues, were calculated using an ANOVA on ranks tests followed by Dunns’s post hoc analysis for each test ran.Bar charts of dominant genera within each sample represent the RA of the top 250 ASVs annotated to the genera level for the entire sample population.

One-way permutational multivariate analysis of variance (PERMANOVA) was used to test for significant differences in beta diversity of samples and provide pair-wise comparisons of beta-diversity. PERMANOVA testing was performed using PAST software using Bray–Curtis similarities. Bray–Curtis similarities were calculated by subtracting the Bray–Curtis dissimilarity of each sample pair from 1. The Bray–Curtis similarity of pup fecal and pup ileal samples to maternal tissues were tested for significant differences within each GM using two-way ANOVAs for the factors tissue type and age using SigmaPlot 14.0. A Holm-Sidak post hoc analysis was used to determine significant differences by pair-wise comparison of each group within each two-way ANOVA analysis via SigmaPlot 14.0 (Systat Software, Inc, San Jose, CA). Spearman correlations between pup fecal and pup ileal Bray–Curtis similarities values were calculated using SigmaPlot 14.0.

The contribution of maternal sources to neonate fecal and ileal composition across time were determined using SourceTracker2 with the Gibbs function and default settings^29^.

### Supplementary Information

Supplementary Figure 1.

Supplementary Figure 2.

Supplementary Figure 3.

Supplementary Figure 4.

Supplementary Figure 5.

Supplementary Figure 6.

Supplementary Figure 7.

## Data Availability

All 16S rRNA amplicon sequencing data supporting this manuscript are available at the NCBI SRA as BioProject PRJNA915408.

## References

[1] Stinson LF, Boyce MC, Payne MS, Keelan JA (2019). The not-so-sterile womb: Evidence that the human fetus is exposed to bacteria prior to birth. Front. Microbiol..

[2] Nagpal R (2016). Sensitive quantitative analysis of the meconium bacterial microbiota in healthy term infants born vaginally or by cesarean section. Front. Microbiol..

[3] Fricke WF, Ravel J (2021). Microbiome or no microbiome: Are we looking at the prenatal environment through the right lens?. Microbiome.

[4] Blaser MJ (2021). Lessons learned from the prenatal microbiome controversy. Microbiome.

[5] Perez-Muñoz ME, Arrieta M-C, Ramer-Tait AE, Walter J (2017). A critical assessment of the “sterile womb” and “in utero colonization” hypotheses: Implications for research on the pioneer infant microbiome. Microbiome.

[6] Ferretti P (2018). Mother-to-infant microbial transmission from different body sites shapes the developing infant gut microbiome. Cell Host Microbe.

[7] Mortensen MS (2021). Modeling transfer of vaginal microbiota from mother to infant in early life. ELife.

[8] Beller L (2021). Successional stages in infant gut microbiota maturation. MBio.

[9] Zhang C (2021). The effects of delivery mode on the gut microbiota and health: State of art. Front. Microbiol..

[10] Hansen CHF (2014). Mode of delivery shapes gut colonization pattern and modulates regulatory immunity in mice. J. Immunol..

[11] Stewart CJ (2018). Temporal development of the gut microbiome in early childhood from the TEDDY study. Nature.

[12] Levi Mortera S (2016). Monitoring perinatal gut microbiota in mouse models by mass spectrometry approaches: Parental genetic background and breastfeeding effects. Front. Microbiol..

[13] Reyman M (2022). Effects of early-life antibiotics on the developing infant gut microbiome and resistome: A randomized trial. Nat. Commun..

[14] Ran X, He Y, Ai Q, Shi Y (2021). Effect of antibiotic-induced intestinal dysbacteriosis on bronchopulmonary dysplasia and related mechanisms. J. Transl. Med..

[15] Sanidad KZ, Zeng MY (2020). Neonatal gut microbiome and immunity. Curr. Opin. Microbiol..

[16] Koenig Jeremy E (2011). Succession of microbial consortia in the developing infant gut microbiome. Proc. Natl. Acad. Sci..

[17] Hughes KR (2020). The early life microbiota protects neonatal mice from pathological small intestinal epithelial cell shedding. FASEB J..

[18] Robertson RC, Manges AR, Finlay BB, Prendergast AJ (2019). The human microbiome and child growth—First 1000 days and beyond. Trends Microbiol..

[19] Sela DA, Mills DA (2010). Nursing our microbiota: Molecular linkages between bifidobacteria and milk oligosaccharides. Trends Microbiol..

[20] Bezirtzoglou E (1997). The intestinal microflora during the first weeks of life. Anaerobe.

[21] Fallani M (2011). Determinants of the human infant intestinal microbiota after the introduction of first complementary foods in infant samples from five European centres. Microbiology.

[22] Asnicar F (2017). Studying vertical microbiome transmission from mothers to infants by strain-level metagenomic profiling. mSystems.

[23] Preidis GA (2015). Composition and function of the undernourished neonatal mouse intestinal microbiome. J. Nutr. Biochem..

[24] Meng Q (2021). Single-cell transcriptome sequencing and proteomics reveal neonatal ileum dynamic developmental potentials. mSystems.

[25] Hart ML (2018). Development of outbred CD1 mouse colonies with distinct standardized gut microbiota profiles for use in complex microbiota targeted studies. Sci. Rep..

[26] Deo PN, Deshmukh R (2019). Oral microbiome: Unveiling the fundamentals. J. Oral Maxillofac. Pathol..

[27] Xiao L (2015). A catalog of the mouse gut metagenome. Nat. Biotechnol..

[28] Vrbanac A (2018). The murine vaginal microbiota and its perturbation by the human pathogen group B Streptococcus. BMC Microbiol..

[29] Knights D (2011). Bayesian community-wide culture-independent microbial source tracking. Nat. Methods.

[30] Schloss PD (2012). Stabilization of the murine gut microbiome following weaning. Gut Microbes.

[31] Albenberg L (2014). Correlation between intraluminal oxygen gradient and radial partitioning of intestinal microbiota. Gastroenterology.

[32] Oliphant K (2021). Bacteroidota and Lachnospiraceae integration into the gut microbiome at key time points in early life are linked to infant neurodevelopment. Gut Microbes.

[33] Alderete TA-O (2021). Early life gut microbiota is associated with rapid infant growth in Hispanics from Southern California. Gut Microbes.

[34] Cabrera-Rubio R (2012). The human milk microbiome changes over lactation and is shaped by maternal weight and mode of delivery. Am. J. Clin. Nutr..

[35] Underwood MA (2015). Human milk oligosaccharides in premature infants: Absorption, excretion, and influence on the intestinal microbiota. Pediatr. Res..

[36] Mirpuri J (2014). Proteobacteria-specific IgA regulates maturation of the intestinal microbiota. Gut Microbes.

[37] Machado Prado MR, Boller C (2019). Discovery and Development of Anti-inflammatory Agents from Natural Products.

[38] Michel C, Blottiere HM (2022). Neonatal programming of microbiota composition: A plausible idea that is not supported by the evidence. Front. Microbiol..

[39] Hart ML (2018). Development of outbred CD1 mouse colonies with distinct standardized gut microbiota profiles for use in complex microbiota targeted studies. Sci. Rep..

[40] Witzke MC (2020). Influence of PCR cycle number on 16S rRNA gene amplicon sequencing of low biomass samples. J. Microbiol. Methods.

[41] Walters WA (2011). PrimerProspector: De novo design and taxonomic analysis of barcoded polymerase chain reaction primers. Bioinformatics.

[42] Caporaso JG (2011). Global patterns of 16S rRNA diversity at a depth of millions of sequences per sample. Proc. Natl. Acad. Sci..

[43] Hammer Ø, Harper DAT, Ryan PD (2001). PAST: Paleontological statistical software package for education and data analysis. Palaeontol. Electron..

