# Supplementary figures and images for: The contribution of maternal oral, vaginal, and gut microbiota to the developing offspring gut
Source: Sci Rep. 2023 Aug 22;13:13660. doi: 10.1038/s41598-023-40703-7 (PMC10444849; doi:10.1038/s41598-023-40703-7)

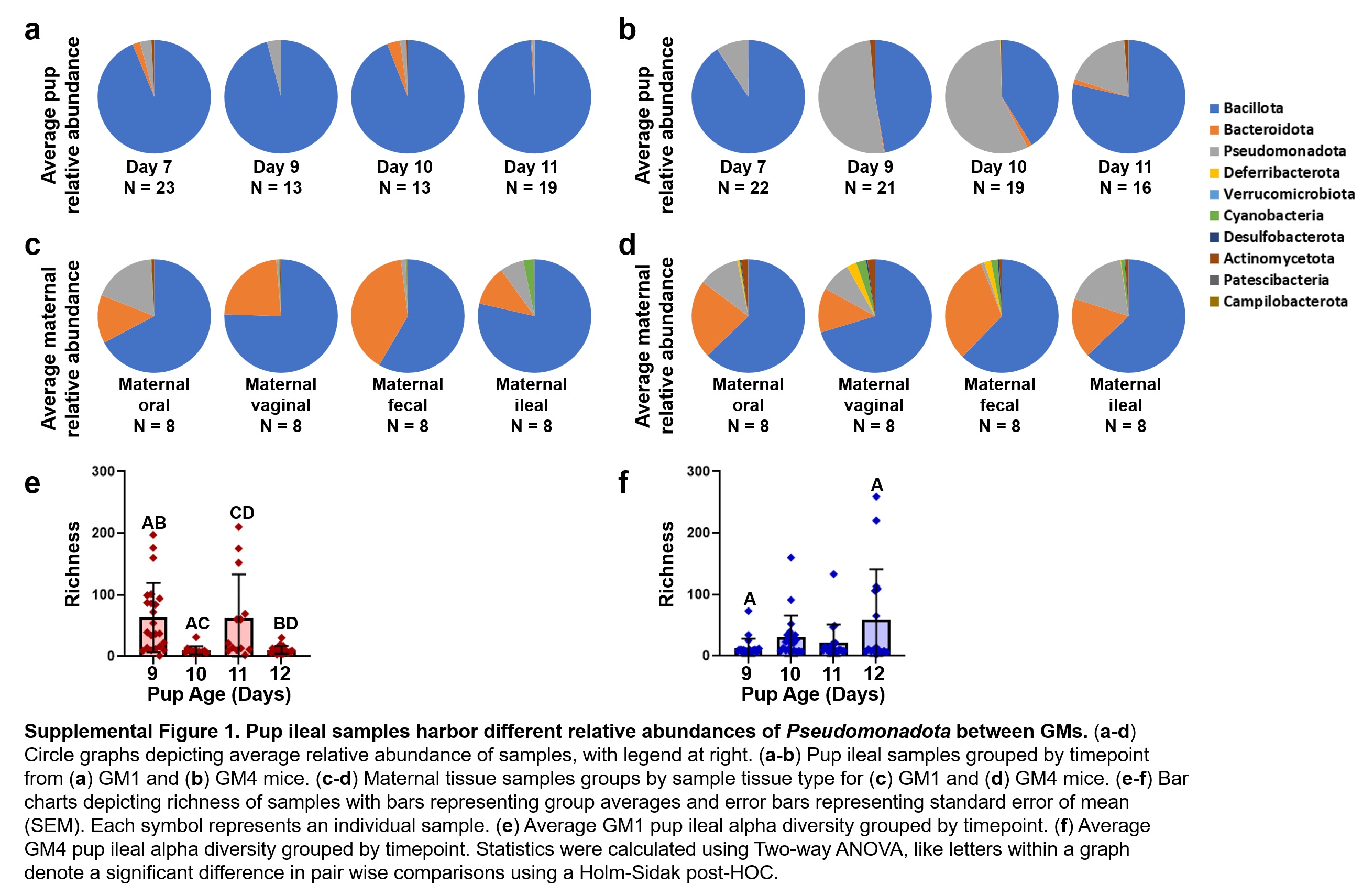

Supplement: Supplementary file 1 — Supplementary Figure 1. [file 41598_2023_40703_MOESM1_ESM.tif]

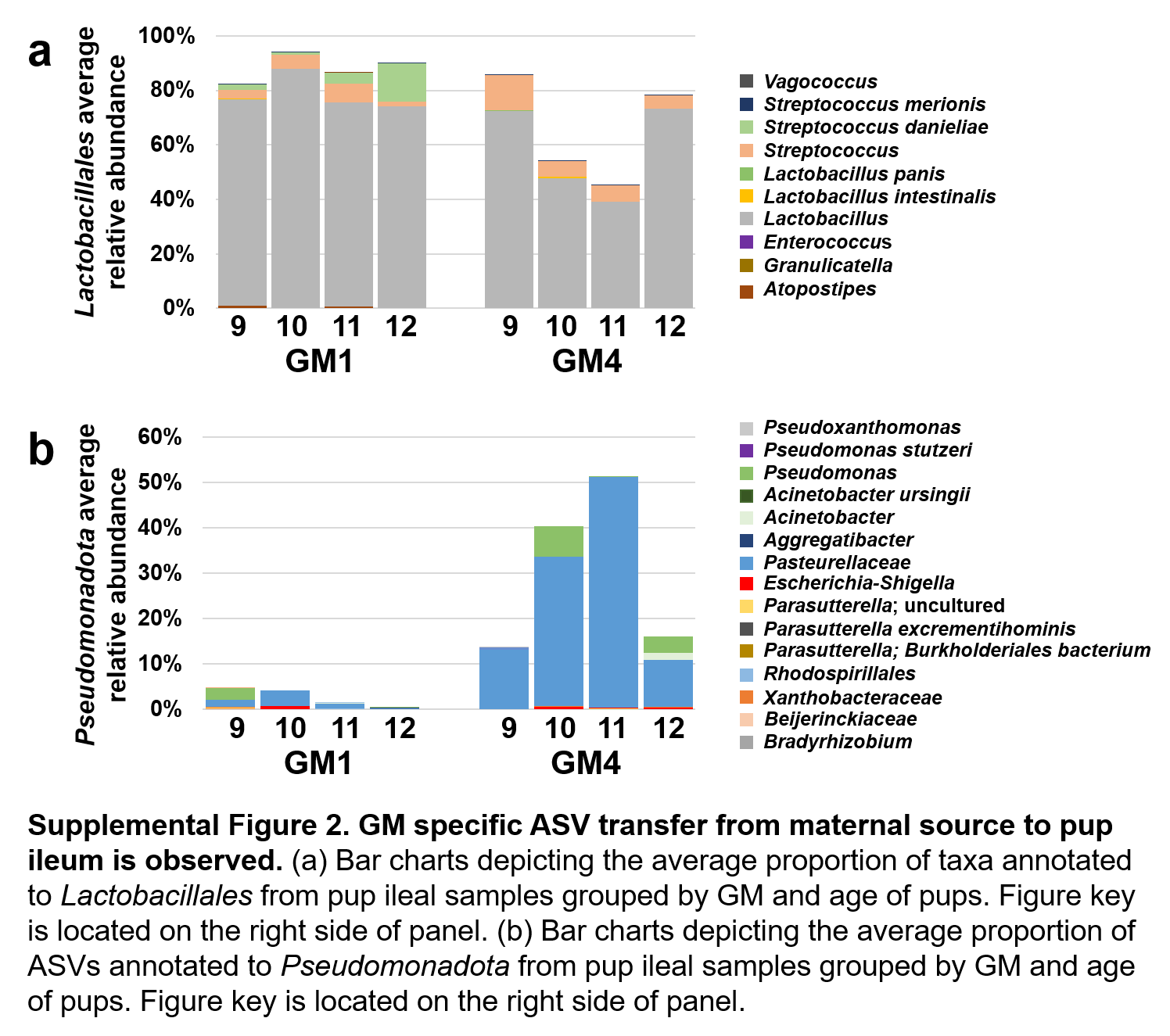

Supplement: Supplementary file 2 — Supplementary Figure 2. [file 41598_2023_40703_MOESM2_ESM.tif]

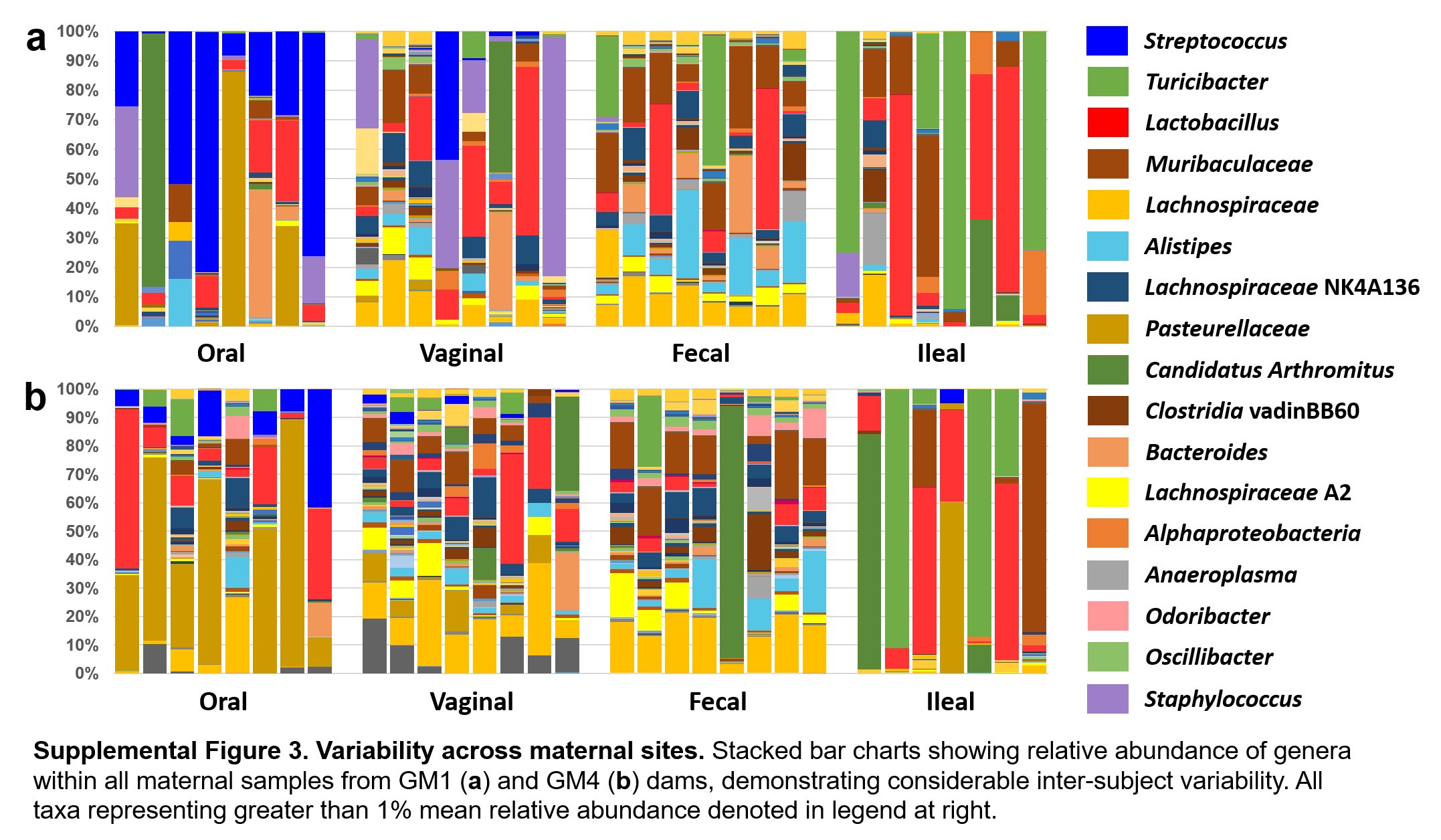

Supplement: Supplementary file 3 — Supplementary Figure 3. [file 41598_2023_40703_MOESM3_ESM.tif]

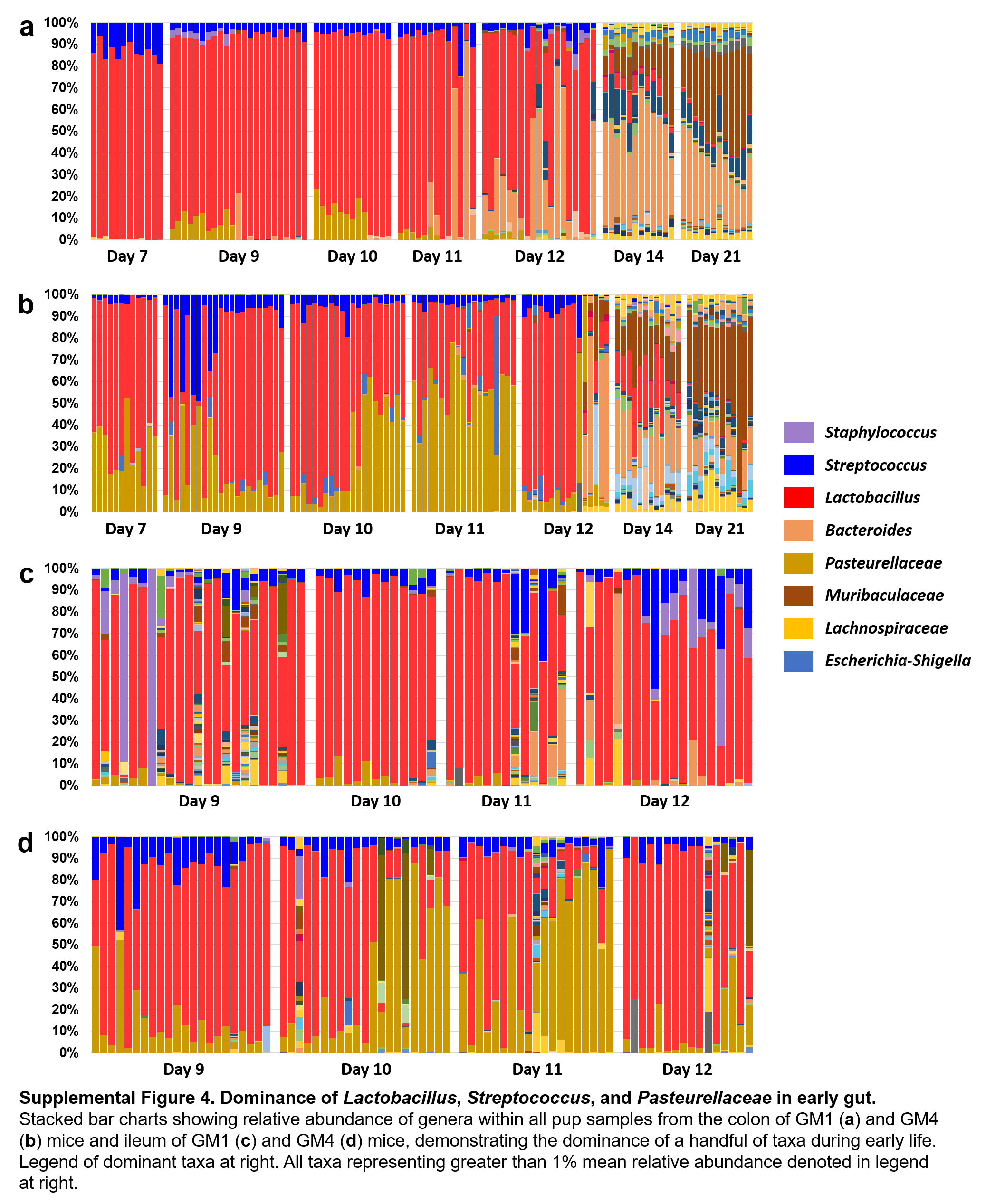

Supplement: Supplementary file 4 — Supplementary Figure 4. [file 41598_2023_40703_MOESM4_ESM.tif]

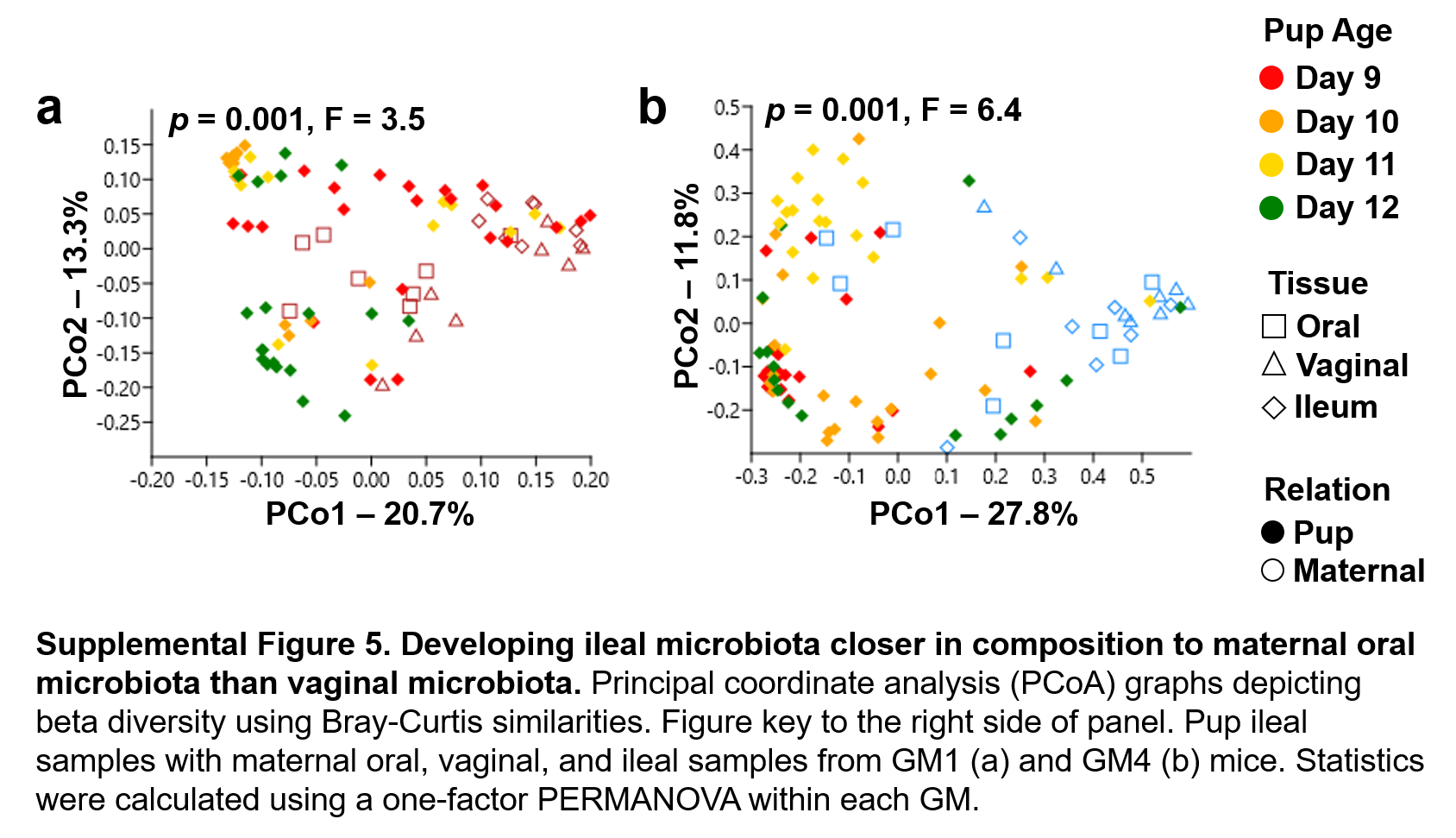

Supplement: Supplementary file 5 — Supplementary Figure 5. [file 41598_2023_40703_MOESM5_ESM.tif]

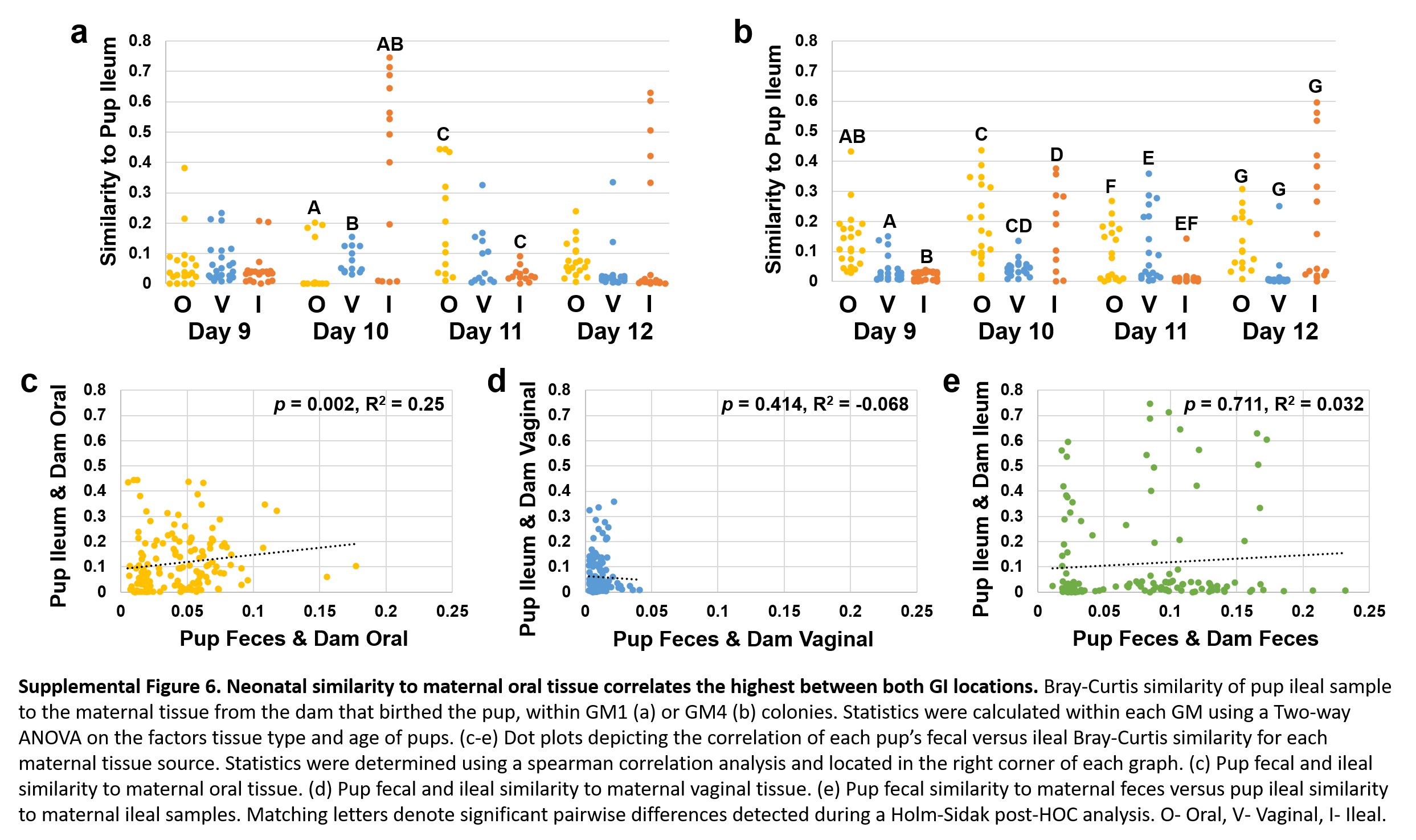

Supplement: Supplementary file 6 — Supplementary Figure 6. [file 41598_2023_40703_MOESM6_ESM.tif]

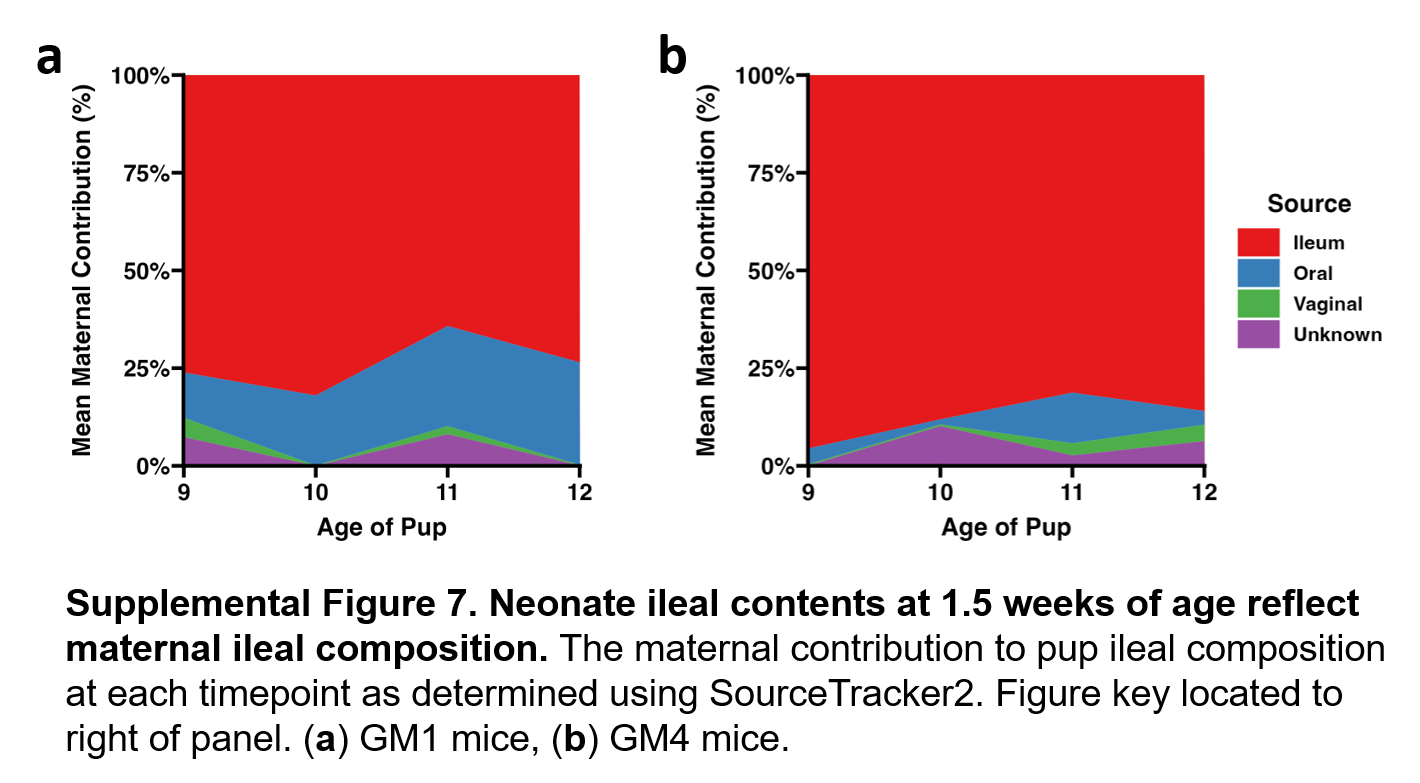

Supplement: Supplementary file 7 — Supplementary Figure 7. [file 41598_2023_40703_MOESM7_ESM.tif]
